# Planned Pregnancy in a Chronic Myeloid Leukemia Patient in Molecular Remission

**DOI:** 10.1155/2012/624590

**Published:** 2012-05-16

**Authors:** Carolina Pavlovsky, Isabel Giere, Germán Van Thillo

**Affiliations:** ^1^FUNDALEU, Centro de Internación e Investigación Clínica Angélica Ocampo, Pte José E. Uriburu 1520, C1114AANP Buenos Aires, Argentina; ^2^IFER, Instituto de Ginecología y Fertilidad, Buenos Aires, Argentina

## Abstract

Excellent response rates and a good quality of life have been observed since the introduction of tyrosine kinase inhibitors (TKIs) in chronic myeloid leukemia (CML) treatment. Consequently, some challenges began to appear in CML women in child-bearing age wishing to become pregnant. Currently, many women around the world are in stable major/complete molecular response MMR/CMR (MMR: <0.1% BCR-ABL/ABL and CMR: undetectable BCR-ABL mRNA by RQ-PCR transcript levels on the international scale). The condition of stable MMR/CMR is linked to a long-term virtual absence of progression to the accelerated and blastic phase and to the possibility of stopping the TKI treatment with the maintenance of a condition of CMR in a proportion of cases. Imatinib teratogenic and prescribing information prohibits the use of it during pregnancy. We describe the case of a 36-year-old female patient with CML in chronic phase who stopped imatinib after 2 years in major molecular response (MMR) to plan a pregnancy. Molecular monitoring by RQ-PCR was performed quarterly. She achieved a safe pregnancy and delivery maintaining an optimal molecular response throughout the pregnancy. Isolated literature reports have been described, but no formal advice has been described at present time.

## 1. Introduction

Tyrosine kinase inhibitors (TKIs) treatment has revolutionized chronic myeloid leukemia (CML) prognosis, improving overall survival to 85% when compared to interferon therapy [1]. Patients who achieve major molecular response (MMR) at 18 months show 95% event free survival (EFS) probability at 72 months when compared with those in complete cytogenetic response (CCyR) but no MMR. This improvement accompanied a better quality of life and consequently some challenges started to grow in CML women in childbearing age who wished to become pregnant. Imatinib teratogenic effects based on animal studies [2] and prescribing information recommends avoiding pregnancy during its treatment [3]. A large description of over 180 women exposed to imatinib treatment during pregnancy has been published [2] and pregnancy and fetal outcome data were reported in 125 (69%) women: 63 patients delivered normal live births (18/63 were under imatinib during their pregnancy), 9 infants were born with fetal abnormalities, 35 (28%) had an elective termination, and 18 (14.4%) showed spontaneous abortion, considering this number as the expected in the normal population. Congenital abnormalities found in 9 born infants are described in Table 1 [4]. Although many of the pregnancies had a successful outcome, the risk of serious malformations due to exposure to imatinib has been the main reason for giving advice to CML female patients to avoid conception. Currently, many women around the world are in stable MMR/CMR (MMR: <0.1% BCR-ABL/ABL and CMR: undetectable BCR-ABL mRNA by RQ-PCR transcript levels on the International Scale) [5]. The condition of stable MMR/CMR is linked with a long-term virtual absence of progression to the accelerated and blastic phase [6–8] and with the possibility of stopping the TKI treatment with the maintenance of a condition of CMR in a proportion of cases. The French STIM study [9] showed that imatinib can be safely discontinued inside a clinical trial in those patients who achieved CMR (>5log reduction in BCR-ABL and ABL levels and undetectable transcripts on quantitative PCR) for at least 2 years. From 100 patients in CMR who stopped imatinib, 39% remained in CMR after discontinuation but a molecular relapse/recurrence of 61% was observed. All patients in molecular relapse/recurrence were retreated with same dose of imatinib (56 reachieved CMR after Imatinib retreatment, 5 pts. did not return to CMR: 4 pts. were continuously free of treatment with a median BCR-ABL level of 0.15% (0.05 to 0.3) at last evaluation and 1 received dasatinib due to a BCR-ABL level of 6.6%, that is, corresponding to a loss of a CCyR), no event as hematologic relapse or progression was observed [10]. So, it is possible to advise a patient who desires a pregnancy to stop imatinib treatment if optimal molecular response has been achieved, considering always appropriate counselling and a very close molecular monitoring, CML women in persistent MMR/CMR that wish to conceive can be well advised not to run any risk for either the mother or the infant [4].

## 2. Case Report

We describe the case of a 38-year-old woman with diagnosis of CML Phi(+) in chronic phase whose main aim was to have a baby and plannified her pregnancy stopping imatinib to be out risk. She achieved a safe pregnancy and delivery maintaining an optimal molecular response through it.

In June 2000, when she was 27 years old, leucocytosis with left deviation was confirmed, during a routine check-up exam. Her physical examination was normal. Peripheral blood (PB) count findings were as follows: white blood counts (WBC) 127 × 10^9^/L with 1% blasts, 18% myelocytes, 12% metamyelocytes, 12% bands, 51% neutrophils, 4% lymphocytes, 2% monocytes, hemoglobin 12.9 g/dL, and platelet count 131 × 10^9^/L. Renal and liver function tests were normal. Cytogenetic and molecular tests confirmed 100% Phi(+) in all analyzed metaphases and presence of BCR-ABL 4 rearrangement by polymerase chain reaction (PCR). Search for HLA compatible donor with her sister was unsuccessful. She had a low-risk Sokal score and her performance status was 0 at diagnosis. She was first treated at another institution and given IFN alpha 5.000.000 U/day associated with cytarabine 30 mg/day for 10 days every 28 days. She obtained complete hematologic and cytogenetic response at 2 and 12 months, respectively, and was maintained over time. In July 2005, she was referred to FUNDALEU, and started imatinib 400 mg/day with excellent tolerance and adherence to medication. She had been monitored by fluorescence in situ hybridisation analysis (FISH) every 6 months with persistence of CCyR. She was advised about imatinib teratogenicity deciding to use contraceptive methods.

**Figure 1:**
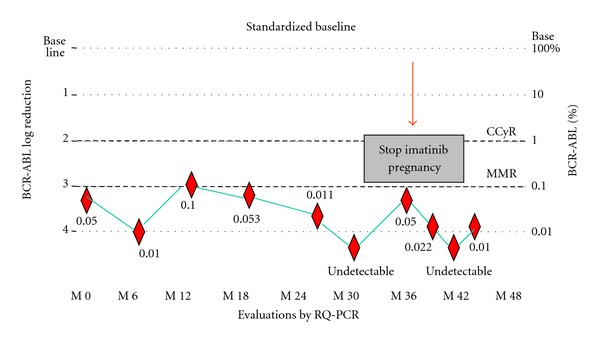
Evolution of molecular response during imatinib treatment.

Since 2007, real-time quantitative PCR (RQ-PCR) started to be performed under the standardization program. In November 2007, she was first tested by RQ-PCR showing achievement of MMR and monitored every 6 months showing a molecular kinetic stability through time with persistent MMR/CMR up to present time. In Figure 1 kinetics of molecular response is described. In September 2009, she expressed her wish to have a baby asking if a future pregnancy could be possible. She received a long explanation of what the literature describes about possible teratogenic effects imatinib causes to the fetus while mother is under treatment and also the possible but unknown risks for progression if interrupting treatment at this stage of the disease. After that she planned the possibility of being carried out. She was then 36 years old, and due to that she was referred to a consultation with a fertility specialist. The first step was to study both, the patient and her husband, in order to detect any abnormality that could potentially delay the achievement of pregnancy before the interruption of imatinib. Basic fertility workup: spermogram, gynaecologic physical examination, hysterosalpingography, basal FSH, LH, estradiol, prolactin and 5 thyroid hormone levels, and cervical and vaginal cultures, as well as transvaginal ultrasound were, between normal limits.

After being told of the risks described above, she signed the informed consent and imatinib was withhold on January 20th, 2010. Planification of monthly visits with blood counts were required associated to RQ-PCR every 3 months. As she had not become pregnant the 2nd month after the interruption of imatinib and with consensus of the hematologist and fertility specialist, it was decided to start ovarian stimulation for intrauterine insemination; although the couple was considered presumably fertile. Ovarian stimulation was performed with 100 mg daily of clomiphene citrate from day 5 to 9 of the menstrual cycle, followed by daily subcutaneous application of 75 I.U. of HMG. When three follicles of 20, 18, and 17 mm. in mean diameter where observed by transvaginal ultrasound, ovulation was triggered with 10000 I.U. of hCG. Thirty-six hours later an intrauterine insemination with 53 × 10^6^ highly motile spermatozoa after swim-up preparation of the semen sample was performed. As a result of the procedure she achieved a singleton pregnancy. She required hospitalization between weeks 23 and 24 for suspected preterm labour, receiving corticosteroids after completion of week 24, to prevent fetal lung maturation. Although treatment was interrupted, she persisted on CHR and MMR during all the pregnancy. Labour started spontaneously in December 2010, at week 39, requiring an emergency caesarean section because of fetal distress. A healthy girl was born, with Apgar Score of 9/10, weight of 3.010 grs, and measuring 49 cm in length and 33 cm of cephalic diameter. Negative DAT and physiologic jaundice. Patient's molecular evaluation after delivery showed MMR persistency, so breastfeeding was permitted. Having breastfed for 3 months, she reinitiated imatinib 400 mg day on April 2011. The girl's growth and development had been normal to date.

Data about imatinib and breastfeeding in humans is still limited and the effects of chronic exposure of infants to imatinib are not known. Its metabolites are excreted in the milk of female rats when given at 100 mg/kg daily with higher concentrations than in plasma. Substantial accumulations of imatinib into breast milk were observed when measured in women taking imatinib postpartum, and due to possible adverse reactions, breastfeeding should be not be permitted [11].

## 3. Discussion

The issue on how to give advice for a planned parenting is still a challenging situation. It is reasonable for only those women who had achieved optimal molecular responses to be the ones who could have the possibility to withhold the drug with a minimum risk during a period of time. Some questions have not answers yet, such as the time to wait between discontinuation of the drug and conception, where some authors suggest it may be reasonable to consider a wash-out period of a few days before conception [9]. Goldman reported that once achieving CCyR and MMR, it might be possible to stop imatinib for a period of time to allow the patient to conceive and carry the child without exposure to the drug but being aware of the risk of progression this situation can bring [12]. Although it is unlikely for patients in persistent MMR/CMR to require treatment, if molecular progression is observed, treatment must be established immediately. Nonteratogenic treatments during pregnancy are not well defined but leukapheresis [13], hydroxyurea, and interferon alfa [14] in the second or the third trimester could be a safe option. At present time, there is no formal advice in the literature on how to manage these situations but it must depend on the response the patient had achieved. It is not clear if CML patients in MMR/CMR can stop imatinib and still be safe, being of great risk to withhold therapy to patients without persistent optimal molecular response.

In this new TKI treatment era, female patients of child-bear age not only aim to achieve optimal treatment responses to be protected from relapse but also consider the possibility to interrupt treatment to plan a pregnancy. Achievement of CMR for at least 2 years seems to be a safe timepoint to plan a patient discontinuation of imatinib.

Several aspects before interruption must be considered: a continuous CMR and a very strict molecular and clinical patient followup. This case report is not a recommendation but the management of a planned pregnancy in a CML patient.

## Figures and Tables

**Table 1:** Congenital defects in 9 infants borned after maternal exposure to imatinib.

Infant	Quarterly exposure	Defect
1	First	Meningocele (stillborn at week 34)
2	First	Premature closure of the skull sutures (craniosynostosis)
3	First	Hypoplastic lungs, exomphalos, duplex left kidney, absent right kidney, hemivertebrae, and a right shoulder anomaly
4	Unknown	Exomphalos, right renal agenesis, and hemivertebrae
5	First	Exomphalos and scoliosis
6	First	Communicating hydrocephalus, cerebellar hypoplasia, atrial septal defect, overriding aorta, ascitis, pericardial effusion
7	First	Hypospadias
8	First	Hypospadias
9	First	Pyloric stenosis
